# Large-scale analysis of the N-terminal regulatory elements of the kinase domain in plant Receptor-like kinase family

**DOI:** 10.1186/s12870-024-04846-7

**Published:** 2024-03-06

**Authors:** Qiong Fu, Qian Liu, Rensen Zhang, Jia Chen, Hengchang Guo, Zhenhua Ming, Feng Yu, Heping Zheng

**Affiliations:** 1 Bioinformatics Center, Hunan University College of Biology, Hunan, 410082 China; 2 Shenzhen H-Great Optoelectronic Co. Ltd, Shenzhen, 518110 China; 3 State Key Laboratory for Conservation and Utilization of Subtropical Agro-bioresources, College of Life Science and Technology, Guangxi University, Nanning, 530004 China

**Keywords:** Juxtamembrane segment, N-terminal regulatory element, Receptor-like kinase, Genome-wide analysis, Phosphorylation, Posttranslational modification

## Abstract

**Background:**

The N-terminal regulatory element (NRE) of Receptor-like kinases (RLKs), consisting of the juxtamembrane segment in receptor kinases (RKs) and the N-terminal extension segment in RLCKs, is a crucial component that regulates the activities of these proteins. However, the features and functions of the NRE have remained largely unexplored. Herein, we comprehensively analyze 510,233 NRE sequences in RLKs from 528 plant species, using information theory and data mining techniques to unravel their common characteristics and diversity. We also use recombinant RKs to investigate the function of the NRE in vitro.

**Results:**

Our findings indicate that the majority of NRE segments are around 40–80 amino acids in length and feature a serine-rich region and a 14-amino-acid consensus sequence, ‘FSYEELEKAT[D/N]NF[S/D]’, which contains a characteristic α-helix and ST motif that connects to the core kinase domain. This conserved signature sequence is capable of suppressing FERONIA’s kinase activity. A motif discovery algorithm identifies 29 motifs with highly conserved phosphorylation sites in RK and RLCK classes, especially the motif ‘VGPWKpTGLpSGQLQKAFVTGVP’ in LRR-VI-2 class. Phosphorylation of an NRE motif in an LRR-VI-2 member, MDIS1, modulates the auto-phosphorylation of its co-receptor, MIK1, indicating the potential role of NRE as a ‘kinase switch’ in RLK activation. Furthermore, the characterization of phosphorylatable NRE motifs improves the accuracy of predicting phosphorylatable sites.

**Conclusions:**

Our study provides a comprehensive dataset to investigate NRE segments from individual RLKs and enhances our understanding of the underlying mechanisms of RLK signal transduction and kinase activation processes in plant adaptation.

**Supplementary Information:**

The online version contains supplementary material available at 10.1186/s12870-024-04846-7.

## Background

**Fig. 1:**
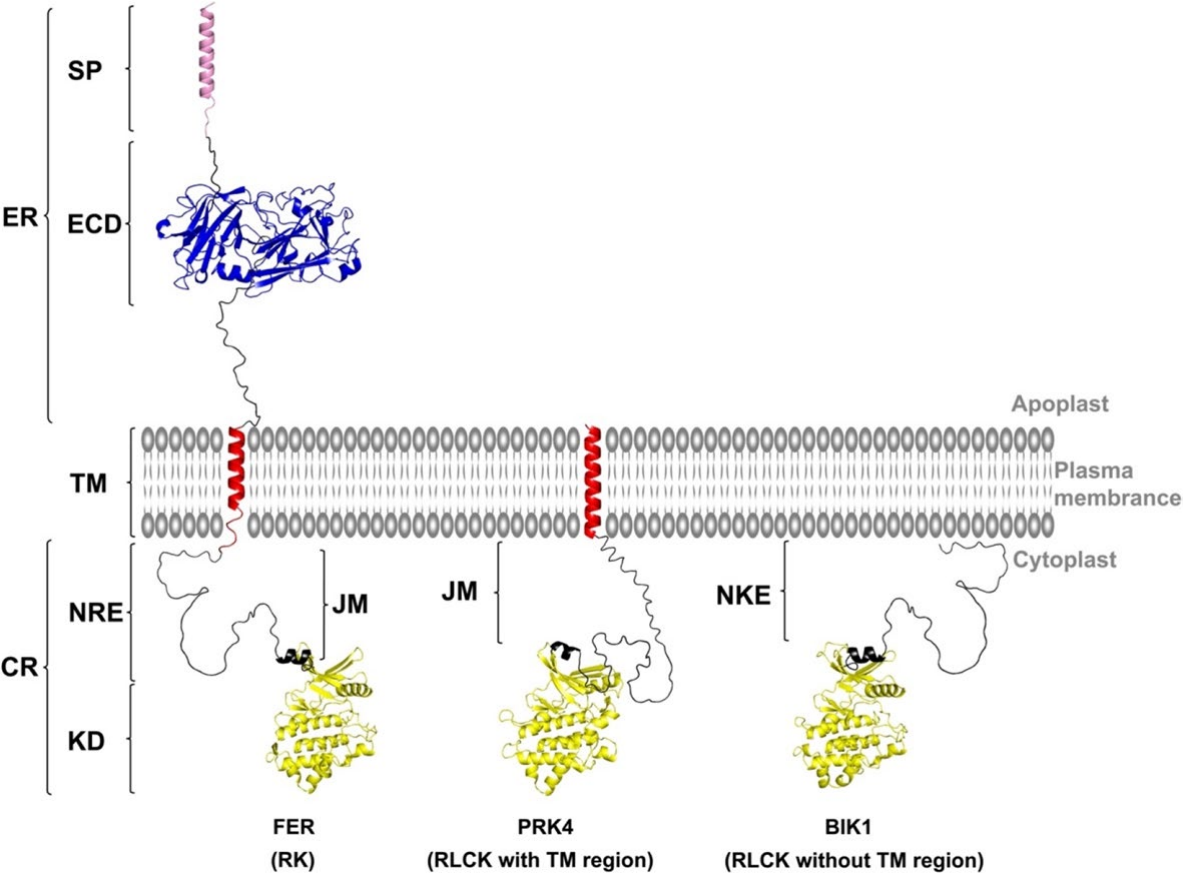
Architecture illustration of plant receptor kinases (RKs) and receptor-like cytoplasmic kinases (RLCKs). RKs (schemes FER) consist of an extracellular ligand-binding domain (ECD, blue shapes); a single transmembrane helix (TM, red cylinders); and a cytoplasmic kinase domain (KD, yellow shapes). Disordered loop regions (dark lines) connect the different domains. RLCK with TM region (schemes PRK4) are consisted of a TM region and a cytoplasmic region (CR), but lack an ECD or have a short unstructured extracellular region (ER). RLCK without TM region (schemes BIK1) lacks both ER and TM region. The juxtamembrane segment (JM) with an α-helix in the C-terminal (dark bold) connects the TM with the KD in RKs and RLCKs with TM region. The NKE is the N-terminal sequence above the kinase domain in RLCK without TM region. JM and NKE are collectively known as NRE. SP: Signal peptide. FER: FERONIA (AT3G51550.1); PRK4: Pollen Receptor Like Kinase 4 (AT3G20190.2); BIK1: Botrytis-Induced Kinase1 (AT2G39660.1)

The Receptor-like kinases (RLKs) family is the largest group of plant kinases responsible for transmitting signal across the plasma membrane. They regulate various signaling pathways related to plant growth and development, perception of external signals, and response to adverse environments. The earliest description of RLK dates back to the 1990 study that described a set of kinases from maize with characteristics similar to receptor tyrosine kinase (RTK) in animals [1]. Typical receptor kinase (RKs) contain a larger extracellular domain (ECD) and a transmembrane helix on top of the cytoplasmic kinase domain, although some lack extracellular ligand-binding domains and are referred to as Receptor-like Cytoplasmic Kinases (RLCKs) [2]. Most RLCKs primarily consist of a Serine/Threonine kinase domain, while others additionally feature predicted signal sequences and transmembrane domain that resemble ECDs [3]. However, these presumed ECDs lack identifiable protein domains and consist of short extracellular sequences (See Results section and Fig. 1). Based on phylogenetic analysis of kinase domains in *Arabidopsis thaliana*, RKs and RLCKs form a large family of plant kinases [4]. Evidence from Expression Sequence Tag (EST) analysis suggests that the RLK gene family emerged early in the evolution of land plants, before the diversification of land plants from their aquatic ancestors [5].

Further research indicates that the RLK family in plants shares similarities with the gene product of *Pelle* from *Drosophila melanogaster* and the Interleukin Receptor-associated Kinases (IRAKs) from mammals [6]. Although *Pelle* and IRAKs in animals are much less common than their RLK counterparts in plants, their similarities suggest a common ancestor from the evolutionary perspective [4]. These three groups form the RLK/Pelle monophyletic clade of kinases according to Shiu protein kinase classification scheme, which subdivides the plant RLK family into 65 classes [7]. The most frequently observed classes in the plant RLK family are RLCKs (20 classes) and Leucine-rich repeat (LRR) kinases (23 classes). Due to extensive gene expansions and functional divergence in response to various environmental signals, the RLK family has undergone highly complex evolutionary changes [8].

Structural topology and domain organization of RKs typically encompass an ECD responsible for sensing various external signals, a single transmembrane region, and a cytoplasmic kinase that activates downstream events. RKs are versatile in sensing a wide range of external stimuli, including partner proteins, peptides, carbohydrates, and organic small molecule. Based on the extracellular domain, the most prominent classes of RK are the S-domain, LRR, and Epidermal growth factor-like repeat class [9]. The S-domain class has been extensively studied in Brassicaceae, where it plays a critical role in preventing self-pollination through the self-incompatibility-locus glycoproteins (SLGs) [10]. LRR kinases play various roles in plant signal transduction processes, benefiting from tandem repeats of highly conserved β-sheets that facilitate extensive protein-protein interactions [11]. Additionally, RLCKs can associate with RKs to regulate biotic and abiotic processes [12]. RLCKs act as common signaling nodes that link RKs to downstream signal output [13]. A phosphorylation relay has been proposed in which RKs phosphorylate RLCKs upon ligand perception, and the RLCKs further phosphorylate major downstream signaling components [14].

The region between the transmembrane region and the cytoplasmic kinase in members of the RLK family is referred to as the juxtamembrane (JM) segment, which is intrinsically disordered [15]. The intrinsic disorder of the JM segment plays a critical role by allowing conformational flexibility, which enables proteins to switch between active and inactive states [16]. Additionally, it promotes interactions with various binding partners that facilitate RK’s fine-tuning of cellular responses. Furthermore, this intrinsic disorder allows these regions to function as flexible platforms for modifications, which can further regulate protein activity and function [16]. In the epidermal growth factor (ErbB-1) receptor kinase, deleting the JM segments caused a severe loss of tyrosine phosphorylation [17]. In the Eph receptor kinase (EphB2), the two conserved tyrosines (Y605 and Y611) in the JM segment regulate the kinase autophosphorylation [18, 19]. In type I transforming growth factor (TGF-β) receptor kinase, phosphorylation of the JM segments prevented the binding of PKBP12 and activated the kinase activity of TGF-β [20, 21]. In *Arabidopsis* and rice, the JM segments of CERK1 and other LysM-RLKs play a crucial role in the chitin-induced signaling process [22], and phosphorylation of this JM segment activates the mechanism to protect the plant against microbial infection [23]. The *Os*WAK11-mediated inhibitory phosphorylation occurs within an STS motif (Ser751-Thr752-Ser753) localized to the JM of *Os*BRI1 found in most monocots [24]. In RLCKs lacking a transmembrane region, 95% of the N-terminal sequence located in the upper part of kinase domain could not be associated with any known domains according to the Pfam database, suggesting intrinsic disorder (Our observation). This observation was further supported by AlphaFold. Since RLCKs lacking a transmembrane region do not possess a JM segment by definition, we have classified the N-terminal sequence as NKE. The NKEs share many typical regulatory roles as the JM segments in RKs, as post-translational modifications (PTMs) and conformational changes of the NKE can also activate or inhibit RLCK activities [25]. The NKE region of the BIK1 protein includes ten phosphorylation sites that can be phosphorylated by SIK1 to increase BIK1’s stability [25]. In this study, we collectively refer to JM and NKE sequences as putative N-terminal regulatory elements of the kinase domain (NRE).

It is increasingly apparent that the NRE is a crucial component in correlating kinase activation with phosphorylation and other PTMs of cytoplasmic regions in RKs and RLCKs. Despite their critical importance, systematic studies on NREs have been limited, mainly due to the conformational flexibility exhibited by this segment. In this study, we conducted a comprehensive investigation into the sequence characteristics of NRE segments from various perspectives, with the aim of uncovering their potential functions within the plant RLK family, utilizing data mining and structural information. Our dataset provides a foundation for further research on NRE segments in individual RKs and RLCKs. By gaining a deeper understanding of NREs, we can uncover the underlying mechanisms and structural-functional relationships involved in RK and RLCK kinase activation and the signal transduction process in plants.

## Results

### Occurrence of putative N-terminal regulatory elements of the kinase domain in the plant RLK family

**Fig. 2:**
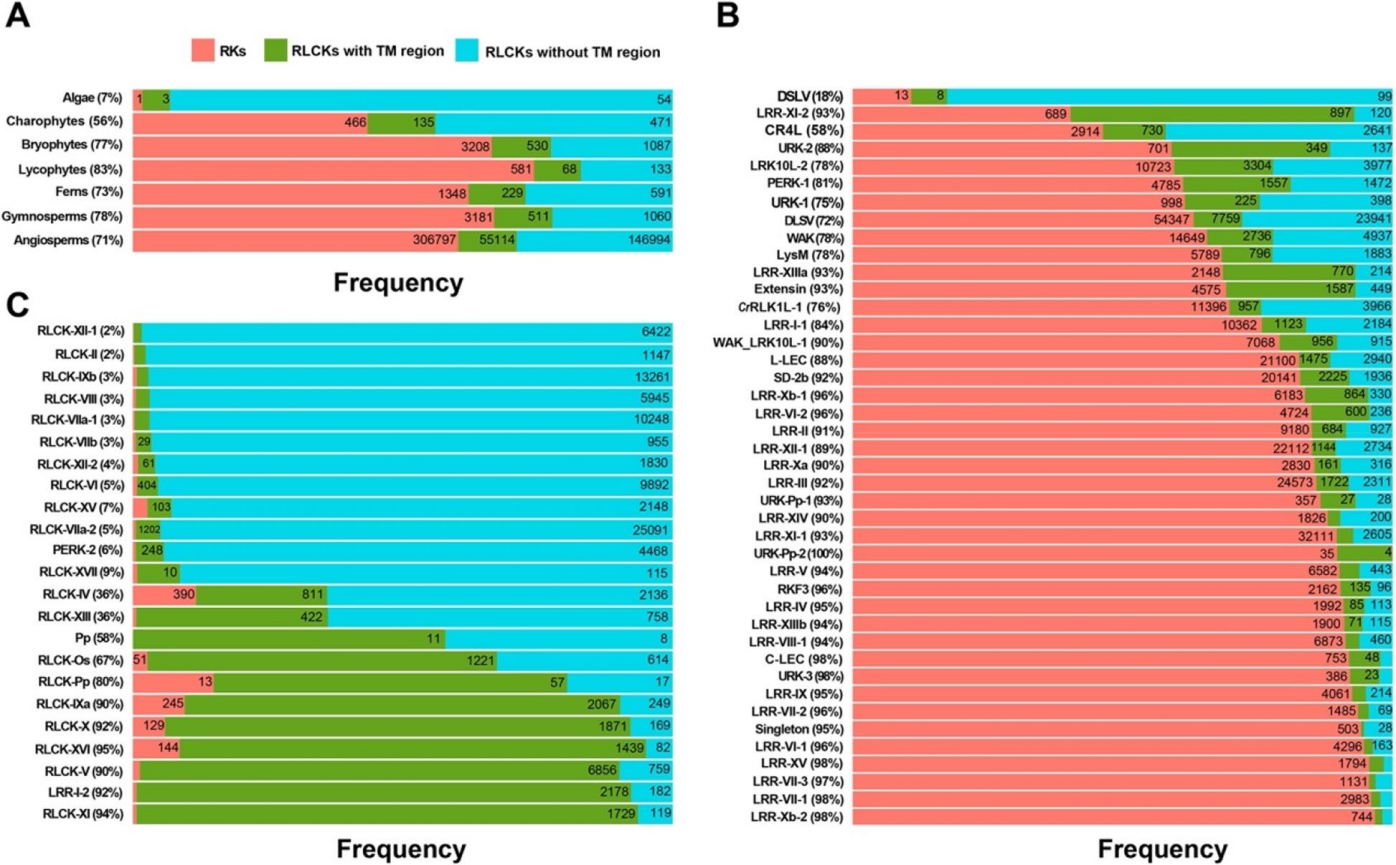
The occurrence of NRE segments in the RLK family. **A**-**C **The emergence of NRE segments in different plant groups (**A**) , RKs classes (**B**), and RLCKs classes (**C**) based on the kinase classification scheme proposed by Lehti-Shiu and Shiu. The red box refers to the topology of RKs with extracellular domains (ECD), transmembrane regions (TM), and intracellular region (CR). The green box refers to the topology of RLCK with limited length of extracellular sequence and without ECD. The cyan box refers to the topology of RLCK without ECD and TM. The percentage refers to the proportion of NRE in RK and RLCK with TM

The presence and distribution of NRE (Fig. 1), which included both JM and NKE, were extensively investigated across various plant groups, comprising 528 plant species, and all RK and RLCK classes, encompassing 510,233 sequences (Fig. 2), as cataloged by the Shiu scheme [4]. Each member of the RLK family was categorized into three distinct topological groups based on the presence or absence of the transmembrane region or ECD and the length of extracellular sequence: RK (including ECD), RLCK with TM region (without ECD and an extracellular sequence of less than 180 bp), and RLCK without TM region (RLCK with NKE) (Our unpublished results). Our non-redundant dataset, identified from the genomic sequence of 528 plant species, revealed the presence of 313,468 RKs, 46,375 RLCK with TM region, and 150,390 RLCK without TM region (Fig. 2). RLCK without NKE was rarely observed, with only < 2000 instances, and was excluded from further analysis in this study. Our dataset comprised a total of 359,843 JM sequences and 150,390 NKE sequences.

In algae, RLCKs without TM region were the primary component, constituting 54 out of a total 58 RKs and RLCKs (Fig. 2A). Charophytes, Bryophytes, Lycophytes, Ferns, Gymnosperms, and Angiosperms exhibited abundant RK sequences, with a relatively lower proportion of RLCK without TM regions and an even lower proportion of RLCK with TM region. This enrichment of RK sequences could be attributed to the accelerated gene duplications coupled with diversifications of RK classes, as compared to RLCK classes, during evolution to develop characteristic vascular bundle and seed features in response to external environments.

The abundance of three topological groups was analyzed in various classes of RKs and RLCKs according to the Shiu classification scheme [8] (Fig. 2B, C). In the RK classes, the majority (72-100%) of the sequences belonged to two topological groups: RKs and RLCKs with TM regions, which indicated the presence of a JM segment, except for DSLV, LRR-XI-2, and CR4L classes (Fig. 2B). The RLCK classes were mainly composed of two topological groups: RLCK with or without TM region (Fig. 2C). RLCK classes with over than 50% of sequences showing a TM region included RLCK-Os, RLCK-Pp, RLCK-IXa, RLCK-X, RLCK-XVI, RLCK-V and RLCK-XI classes (Fig. 2C). However, a few RK classes, such as PERK-2, Pp, and LRR-I-2, were assigned to the topology of RLCK (Fig. 2C). Almost 94% of members of the PEPK-2 class lacked a characteristic TM region. Additionally, more than 90% of members of the LRR-I-2 class possessed a TM region, but their extracellular sequence was exceptionally short, which did not qualify as an ECD according to our topological assignment algorithm. Further investigation revealed that the vast majority (about 72.2%) of the LRR-I-2 class members did not possess any LRR motifs. The classification of the LRR-I-2 class as an LRR class could be due to insufficient data availability in the original RK classification study [8].

### Length and composition of NRE segments in RKs and RLCKs

**Fig. 3:**
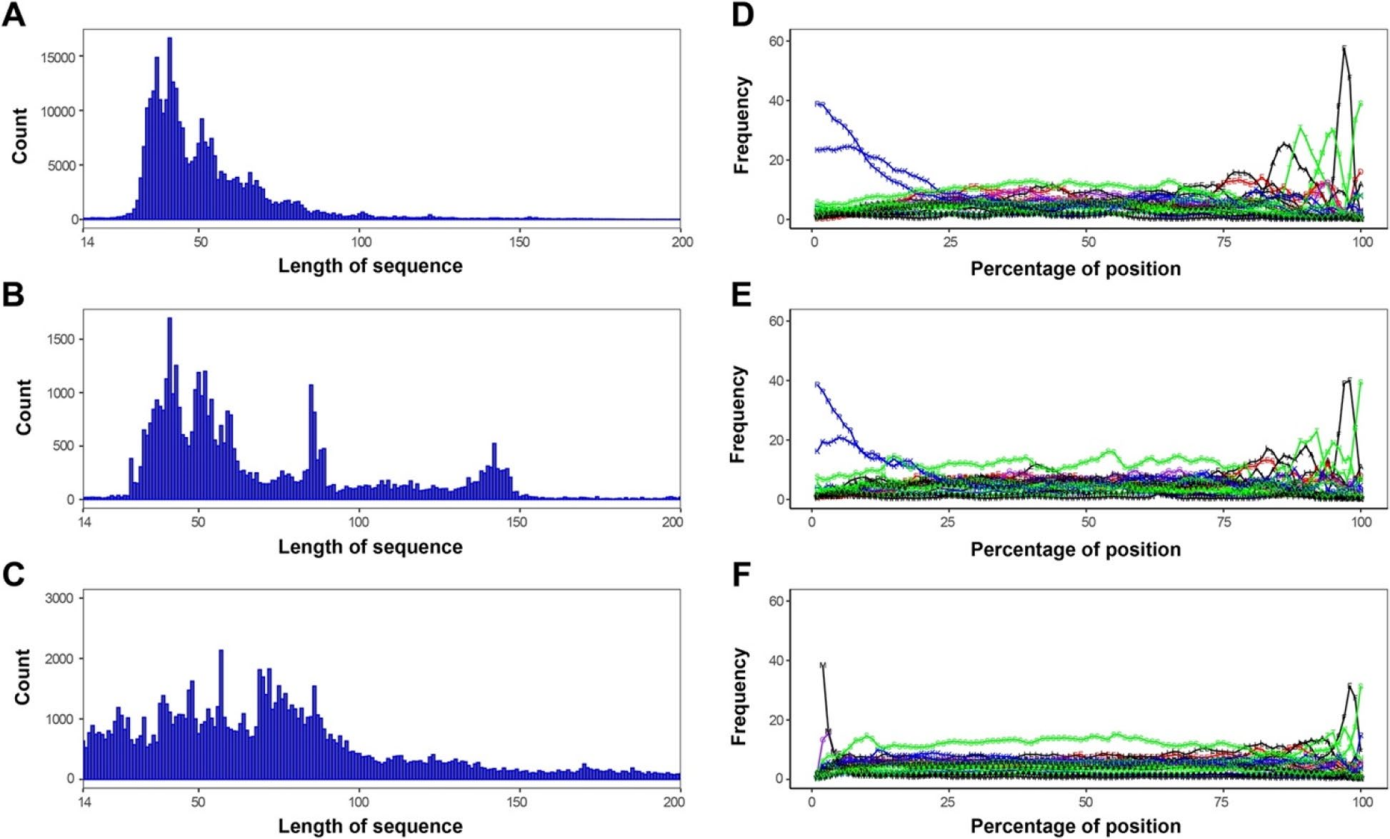
The length distribution and composition of NRE segments in the RLK family. **A**-**C **The length distribution of NRE segments. **D**-**F** The composition of NRE segments. **A**, **D **represent RK sequences. **B**, **E **represent RLCKs with TM region. **C**, **F **represent RLCKs without TM region. The various lengths of different NRE segments were scaled to 200 to align both N-terminal and C-terminal. The composition percentage at a specific position is indicated by the 1-letter code of the amino acid with lines connected along the NRE segment. Hydrophobic, polar, acidic, basic, and neutral residues are shown in black, green, red, blue, and purple, respectively

Analysis of the length of NRE segments indicated that most NRE segments in RKs and RLCKs with TM region had a length between 30 and 100 amino acid residues, with a peak at around 40 amino acids and a secondary peak at about 50 amino acids. However, their abundance decreased as the sequence length increased (Fig. 3A, B). Additional peaks were observed around 80 and 140 amino acids in RLCKs with TM region (Fig. 3B), but the length of NRE segments in RLCKs without TM region did not show any noticeable peak between 14 and 100 amino acids (Fig. 3C). The distribution of NRE sequence length varied significantly among different classes of RKs and RLCK. The typical NRE sequence length was around 50 amino acids in most classes, but some classes, such as LRR-V, RLCK-VI, and RLCK-IXb, exhibited a flattened distribution of NRE sequence length in the range of 60–200 amino acids (Fig. S1).

Regarding the composition of amino acids in the NRE segment, RKs and RLCKs with TM region had similar compositions, with their N-terminal sequences dominated by basic residues arginine (R) and lysine (K) (almost 60%) that gradually decreased to ~ 10% for the first 20% of the NRE sequence (Fig. 3D, E). In contrast, their C-terminal sequences had several characteristic residues with a single peak above 20% abundance in composition: alanine (A), threonine (T), asparagine (N), phenylalanine (F), and serine (S), in that order (Fig. 3D-F). The observation of multiple peaks indicated the presence of a common C-terminal sequence for all NRE segments for RKs and RLCKs sequences. The whole NRE sequence had a consistently high serine (S) composition, with a broad plateau above 10% between 20% and 80% of the NRE sequence, indicating the versatility of NRE as a substrate that was subject to serine phosphorylation. Other amino acids that exhibited peaks above 10% in the composition included glutamic acid (E) and leucine (L), both of which had a primary peak at about 80% along the NRE sequence and a secondary peak at about 30-40% along the NRE sequence.

### Sequence features of NRE segments from different classes of RKs and RLCKs

**Fig. 4:**
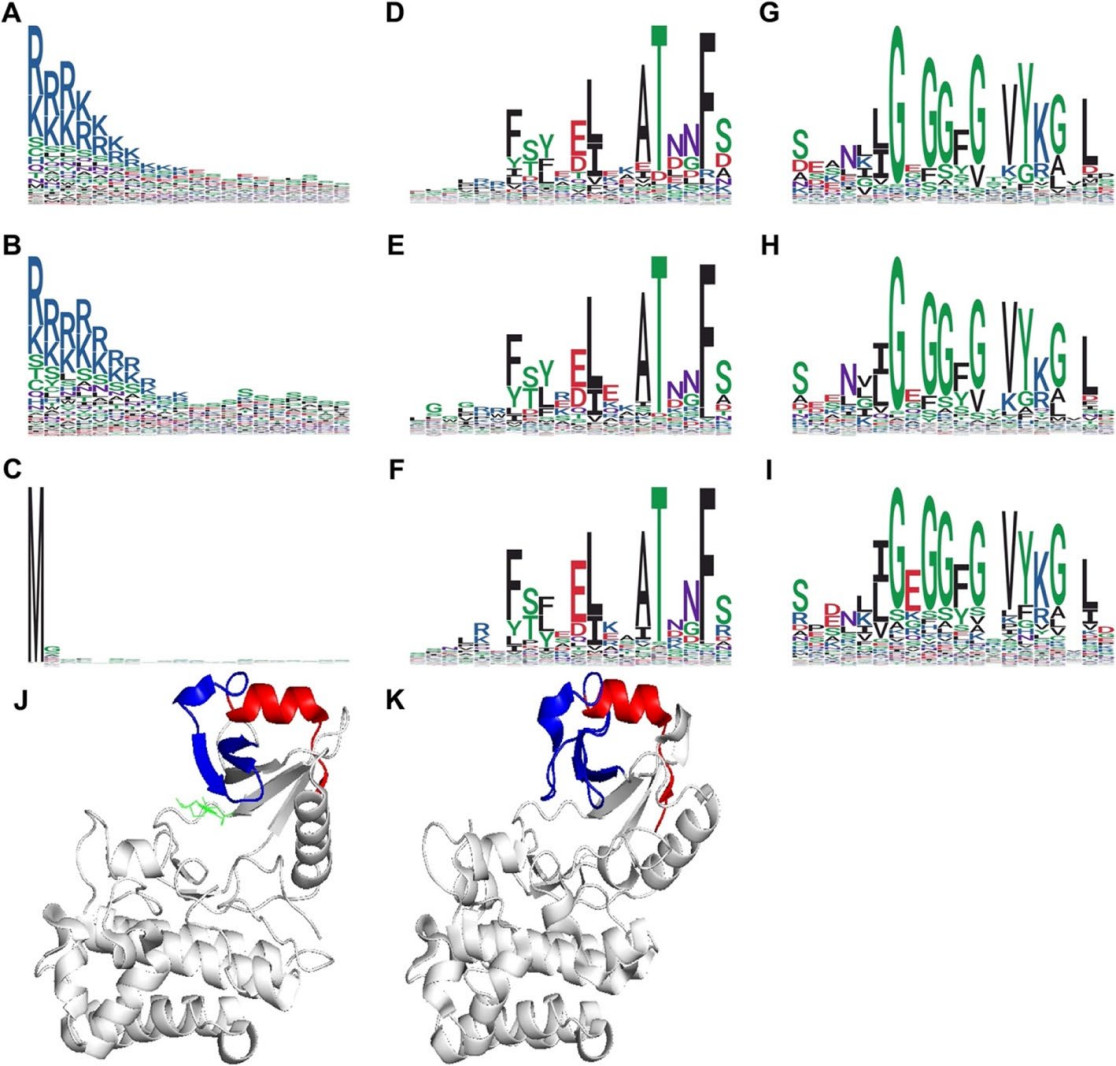
The characteristic sequences of NRE segments in the RLK family. **A**-**C **The characteristic sequences at the N-terminal of NRE segments. **D**-**F **The characteristic sequences at the C-terminal of NRE segments. **G**-**I **The characteristic sequences at the N-terminal of the kinase domain. **A**, **D**, **G **represent RK sequences. **B**, **E**, **H** represent RLCK sequences with TM region. **C**, **F**, **I **represent RLCK sequences without TM region. **J**, **K **Structure of the kinase domain with partial NRE segment (C-terminal) of representative RK (FER, 7XDX) and RLCK (BSK8, 4I93) proteins, with the C-terminal of the NRE segment highlighted in red, and the N-terminal of the kinase domain highlighted in blue. Hydrophobic, polar, acidic, basic, and neutral residues are shown in black, green, red, blue, and purple, respectively

WebLogo analysis of the amino acid profiles of the NRE sequence revealed the presence of high ‘RK’ content at the N-terminal in RKs and RLCKs with TM region, and the presence of a conserved 14-amino acid ‘FSYEELEKAT[D/N]NF[S/D]’ at the C-terminal for all RKs and RLCKs sequences (Fig. 4), in agreement with the composition analysis (Fig. 3D-F). Additionally, a characteristic neutral residue glutamine (Q) / asparagine (N) was observed at the N-terminal of some RK and RLCK classes defined in the Shiu classification scheme (Fig. S2). For example, Q was observed at the N-terminal of classes LRR-I-2, LRR-IV, LRR-VIII-1, LRR-XIIIb, RLCK-VIIa-2, URK-3, and URK-Pp-2, while N was observed at the N-terminal of classes LRR-I-2, LRR-VI-2, LRR-VII-1, LRR-XIIIb, and Pp.

The conserved 14-amino acid ‘FSYEELEKAT[D/N]NF[S/D]’ immediately adjacent to the N-terminal of the kinase domain started with an amphiphilic α-helix (Fig. 4J, K). This α-helix was followed by one conserved motif that was described as helix-capping element: an ST motif bearing the typical signature Thr-X-X-Phe (TxxF), which was characterized by hydrogen bonds from the T hydroxyl group to the F backbone amine and from the T backbone carbonyl group to the S/D (-1) backbone amine. The threonine at -5 position was conserved among all RK and RLCK NRE segments, except for LRR-III, LRR-VI-2, LRR-XI-2, LRR-VII-1, RLCK-II, and Singleton classes (Fig. S3). In these classes, threonine − 5 was primarily substituted by leucine, cysteine, aspartic acid, and phenylalanine, which formed a slightly variant ST motif.

In the middle of the NRE segment, serine-rich regions were found up to 25% abundance beginning at 20% of the NRE sequence and ending at 60% of the NRE sequence, especially in classes C-LEC, DSLV, Extensin, LRR-I-2, LRR-IV, LRR-IX, LRR-VII-1, LRR-VIII-1, LRR-Xa, LRR-Xb-2, LRR-XIIIa, LRR-XIV, RLCK-IV, RLCK-V, RLCK-XVI, Singleton, URK-1and URK-Pp-2 (Fig. S3). A single leucine-rich region was found close to the C-terminal of the NRE segments in LRR-III, LRR-VII-2, LRR-Xa, LRR-Xb-1, LRR-XIIIa, RLCK-IXa classes. An additional leucine-rich region was also found in the middle of the NRE segments in LRR-II, LRR-VI-1/2, LRR-XIIIb, and WAK classes (Fig. S4).

### The characteristic C-terminal sequence of the NRE segment modulated FERONIA kinase activity

**Fig. 5:**
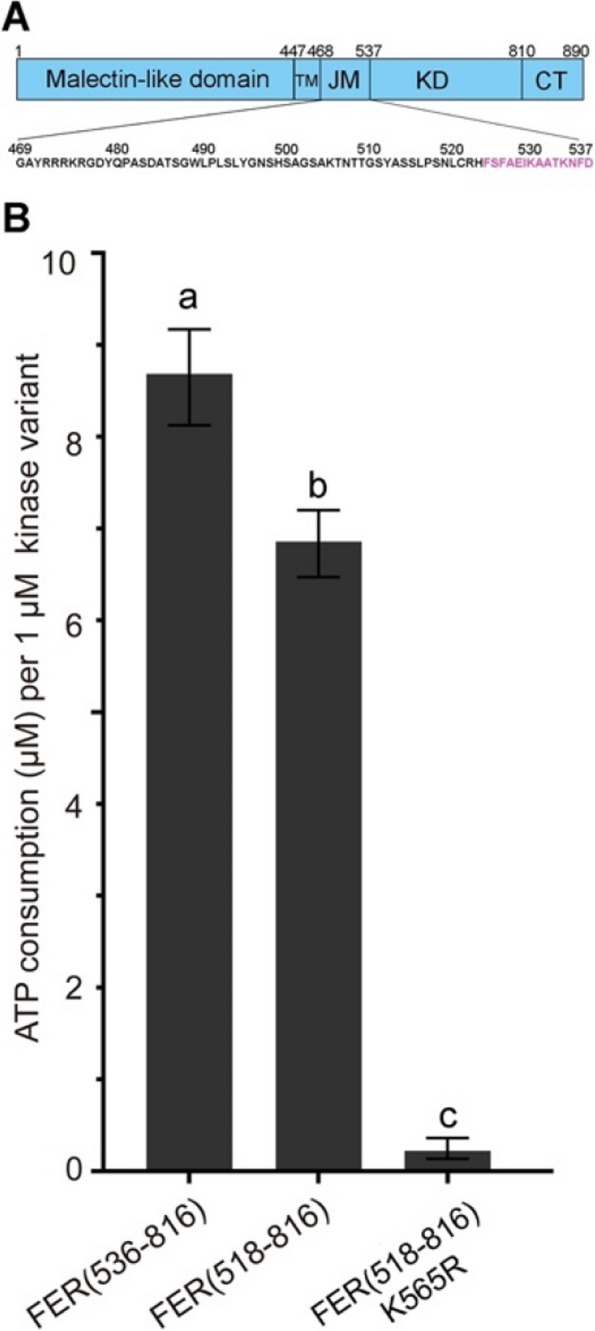
The characteristic C-terminal sequence of the NRE segment modulates FER kinase activity. **A **Schematic representation of the FER protein. The amino acids marked in purple represent the C-terminal sequence of the NRE segment. **B **Effects of C-terminal sequence of the NRE segment on the kinase activity of FER, data shown are mean ± SD of three biological replicates. *P* -values < 0.05 indicate significant differences (two-way ANOVA with post-hoc Tukey HSD test)

To evaluate the relevance of the universal α-helix and ST motif of the NRE segment, we selected a well-studied RK found in the model plant *A. thaliana*, FERONIA (FER, AT3G51550), and quantified its kinase activity by measuring the amount of ATP consumption. Two constructs were prepared to recombinantly express the kinase domain of FER: one with the α-helix and ST motif (residues 518–816) and one without the sequence (core kinase, residues 536–816) (Fig. 5A). The FER K565R mutant was used as a negative control as it did not exhibit any kinase activity. Our findings indicated that the FER^KD^ core kinase (residues 536–816) consumed ATP faster than FER with the NRE segment sequence (residues 518–816) (Fig. 5B). These results suggested that the characteristic C-terminal sequence of the NRE segment could negatively regulate FER’s kinase activity by suppressing its ATPase activity.

### Identification of motifs harboring phosphorylation in the NRE segments

MEME analyzed the NRE segments and identified 65 motifs with a *p*-value threshold of 0.005 and a normalized frequency higher than 1. All motifs that were found in modern embryophytes were also present in early diverging streptophyte algae (Charophytes), while 14 of the 65 motifs were identified in algae (Fig. S5). Out of the 65 motifs, 31 had experimentally determined phosphorylation sites associated with various biological processes such as abiotic stress responses, development, and plant-pathogen interactions in embryophytes (Table S1). Of these phosphorylation motifs, 21 motifs had a single (39%) or double (29%) phosphorylation sites, while the remaining 10 motifs had three to nine phosphorylation sites (32%). The frequency of tyrosine phosphorylation was low, which might be due to its frequent multiphosphorylation (75%) [26]. Interestingly, our analysis revealed the presence of phosphotyrosine sites in four multiphosphorylated motifs out of the 31 motifs analyzed (Table S1).

After scanning NRE sequences for matches to phosphorylation motifs using FIMO, we further screened the most conserved motifs for conservation analysis by examining sequence identity in each specific position of aligned NRE sequences. Out of 31 phosphorylation motifs, 29 showed up to 50% sequence identity (SI) with the matching NRE sequences (Table 1). The highest-scored motif with the mean sequence identity (SI = 87.0%), ‘VGPWKpTGLpSGQLQKAFVTGVP’, was highly conserved in the LRR-VI-2 class (*N*m = 3523, *P* = 3523) and emerged in streptophyte algae (Fig. S5A). The motif showed ubiquitous species presence across virtually all plant groups (from 490 plant species), suggesting the evolutionary conservation of this phosphorylation event. In addition, several motifs with experimentally determined phosphorylation sites were found on several key RK classes involved in plant development and defense of abiotic and biotic stimuli, including *Cr*RLK1L-1, LRR-XI-1, RLCK-XII, and RLCK-VII. Out of the 65 analyzed motifs, the other 32 motifs recorded no experimentally-determined phosphorylation event with similarity above 50%, while 28 still included at least one serine, threonine, or tyrosine that could potentially be phosphorylated (Table 2).

**Table 1:** The identified 29 sequence motifs in NRE segments with experimentally determined phosphorylation sites in classes from Shiu classification (Sequence identity > 50%)

Motif	RK and RLCK classes	Phosphorylation sites	Plant species numbers	The mean SI (%)	Normalized Frequency	*N*m	*N*c	P
VGPWKTGLSGQLQKAFVTGVP	LRR-VI-2	6;9	490	87.0	58.3	3532	5265	3532
KEPLSINVATFEKPL	LRR-Xb-1	5	490	79.4	21.7	3183	7026	4333
TSSEQKSDITDSCSQMILQLHDVYDPNKINVKIKIVSGSPC	PERK-2	1	446	78.9	45.8	2075	4546	2082
SIRGPVVTPTSSPELGTPFTATEAGTSSVSSSDPGTSPFFI	PERK-2	8;11;12	454	76.0	40.8	1856	4546	1870
QKRPQDWQKRNSFSSWLLPLHAGDSSFMSSKSS	*Cr*RLK1L-1	12;14;25;26;30;32;33	397	75.9	2.3	956	13,671	956
SQPKVLRLNLVGSPKKEPE	PERK-2	13	472	73.6	60.0	2719	4546	2729
KVPASPLRVPPSPSRFSMSPKLNRJGSVH	RLCK-IV	5;12;19;27	275	72.9	42.6	627	2572	639
SSGGRSEKPKEEFGSGVQEAEKNKL	LRR-III	6;15	463	70.6	1.6	2587	26,895	2587
PSSASLSFGSSIAAYTGSAKT	Extensin	7	446	70.4	26.8	2369	6342	2383
DDEEEETGCWVKLRFIGSCISSRSKVDSSISGT	RLCK-VIIa-2	28	384	69.9	1.2	1402	22,899	1418
CSSVYHHERAGSSQSGEEGSSGTVRKQSS	RLCK-V	2;3;12;13;15;20;21;23	421	69.8	11.6	1367	7345	1367
SAVELLQKYKKERDELQVERDNALKEAEELRKKQAEASSSH	RLCK-IXb	9;40	341	69.3	6.7	1023	8334	1023
PIRANGADSCTILSDSTIGPESPVKSGRNGMSLWLEGFRRSNVVSASGIP	LRR-I-2	14;22;47	374	67.7	86.6	976	2261	985
SQIPNVSKEIKEDRV	RLCK-V	1;7	485	67.6	38.3	4536	7345	4555
TGSYASSLPSBLCR	*Cr*RLK1L-1	1;3;4;6;7;10	479	66.8	6.6	2818	13,671	2932
SSGWLPLPLYGNSH	*Cr*RLK1L-1	1;2;10;13	470	66.2	5.4	2856	13,671	3683
RRRKKKLSGLNGGYVMPSPLGSSPRSDSSFTKTHSSAPLVG	PERK-1	28;29	175	64.7	3.1	376	7414	376
TGYSSKLLABARYISQTMKLGALGLPAY	LRR-VI-1	5	475	64.6	65.8	2766	4386	2767
KLKZKFFKQNGGLLLQQQJSS	WAK	20	452	63.9	13.2	10,782	19,290	10,858
SSSSKVSSASVPPTPRSE	RLCK-VIIa-2	1;2;3;4;7;8;10;14;17	464	63.7	2.5	3085	22,899	3361
SWPWKLTAFQ	LRR-XI-1	7	495	63.7	3.0	7085	32,872	7205
TKNGBLFSIWNFD	LRR-XI-1	1	289	63.4	1.1	2653	32,872	2668
MGNCLSSRIKAESPSSTGSSS	RLCK-VIIa-2	13;15;17;20;21	399	62.6	1.5	1785	22,899	1806
IGMSNYRSDLDFSGNV	PERK-2	13	393	62.2	33.8	1550	4546	1574
MGCFSCFGSKK	RLCK-VIIa-1	9	486	61.8	15.5	4580	9333	7107
ZELEKMKNQRDEVMEELQMALDQKSSLESQIAESDQMVKEL	RLCK-IXb	29;34	285	61.3	6.7	1019	8334	1028
SZFKASVLEAPDVENEEKSEVDB	RLCK-XII-1	6;19	247	60.2	8.9	684	5924	684
NIFTRKFKGPDVPSTVLKSAPDFCTVYVISKGKJ	RLCK-IXb	30	363	55.7	7.0	1060	8334	1061
PPPPPPPPPMMKSDPYGGQAFSWPHNPP	PERK-1	13;22	254	53.8	3.3	490	7414	602

*SI *Sequence identity, *N*m the number of occurrences of the subject motif in the given RK and RLCK classes, *N*c the number of a given class in the dataset according to either the Shiu classification scheme, *P *the total number of the subject motif in the dataset

**Table 2:** The identified 32 sequence motifs in NRE segments without experimentally determined phosphorylation sites in classes from Shiu classification (Sequence identity > 50%)

Motifs	RK and RLCK classes	Plant species numbers	The mean SI (%)	Normalized Frequency	Nm	Nc	P
LSRNAPPGPPPLCSICQHKAPVFG	PERK-2	489	82.6	69.2	3134	4546	3144
VVVAVKASKEIPKTALVWALTHVVQPGDCITLLVVVPSHSS	PERK-2	467	80.4	55.5	2513	4546	2522
GAVAAEAKRAQANWVVLDKQLKHEEKRCMEELQCNIVVMKR	PERK-2	480	80.0	61.4	2782	4546	2792
PQEIFFDVPAEEDPEVHLGQL	LRR-II	485	78.6	16.8	3658	9996	3658
RSGAPQKVLPIEI	RLCK-VIII	431	78.2	12.4	930	5635	1004
LWGFPRFAGDCASGH	PERK-2	470	78.1	52.1	2364	4546	2374
MRRWLCCTCQV	RLCK-VIII	442	77.9	24.3	1694	5635	1705
ITAPSPLVGLPEFSHLGWGHW	RLCK-V	486	76.9	35.7	4218	7345	4224
DVEAEMERLRLELKQTMDMYSTACKEALDAKQ	RLCK-IXb	350	75.0	7.1	1080	8334	1088
MFCCGGAEEEPYGPPANQYTAPP	RLCK-VIII	387	72.4	11.1	800	5635	832
DIHMVEAGNMV	LRR-IX	466	70.6	51.0	1983	4182	2018
GVLHRVTHPMGYQTKACPDSF	PERK-2	246	70.3	9.2	422	4546	427
DGRLSTDQAKEFYRKSASPLVSLE	LRR-IV	383	69.3	127.0	1220	2096	1221
NGSILLEKLIASCNGKSNPIR	RLCK-XII-2	164	69.0	150.7	1210	1678	1579
GTSIRAMEEEVSKKVDAYVNMLLQSAEECEDEGVSIEVKI	PERK-2	216	68.6	8.0	365	4546	370
YAEVREDWELEYGPH	L-LEC	473	68.4	7.4	8543	23,040	8560
WNPKSRVMGSTRKEVTVFTDI	LRR-XV	321	68.2	95.3	693	1823	694
MGCRCSKLSPC	RLCK-XII-1	464	68.0	38.9	3064	5924	3149
SSDTESENLSLSSASLRFQPWITEYLSS	PERK-2	293	67.5	24.3	1104	4546	1113
LLWALQNFAGKKICILHVHQ	RLCK-IXb	389	65.8	10.9	1656	8334	1661
TIPKRDDHEIDMSRLDVDSMPPPPPPPP	LRR-V	257	65.7	7.1	764	6996	764
YDQLEQAMAEAENSRREAFEESVRRRKAE	RLCK-IXb	421	64.8	9.1	1390	8334	1392
PAQMIPMMGAKFPASSLKEZZVRAYREIE	RLCK-IXb	348	63.7	5.8	882	8334	882
YSRKMKDLKSKKAIYVCZQAPPSCHIWFICKGHLIYTREA	RLCK-IXb	449	63.6	14.0	2132	8334	2135
PSRVVIAYDATKDRNEHELKLTIDNIRMRGDILRGGDTLLV	PERK-2	223	63.0	7.9	361	4546	365
KEDTSSPVGVLEDYFRSSDSETSSSKEPTSDSESQQNSKPASRWHG	RLCK-VI	165	62.8	2.6	339	7655	339
ENXCVQESHLLPVKDKSSEKDSG	RLCK-V	51	60.3	1.7	198	7345	198
YLLICARKKVQAEKVVIEMDDVAKGIVELISQHGITKLVMGAA	RLCK-IXb	447	59.7	13.2	2010	8334	2012
QPTNQMEKVPKEAVVKPKDGHQTESRRMGAIPKPQBEQE	LRR-V	99	58.4	2.7	293	6996	293
LSTSQIIQKQNKSVFRQKSSEAPJLCAACGLRTELY	PERK-2	128	58.2	4.7	215	4546	217
RHLEMYDKIEKFLQDYGNLKP	LRK10L-2	226	56.4	5.3	2476	14,630	2476
LEAJRKAKAAESLAEEEAKQRKEAEEALAK	RLCK-IXb	394	55.5	9.5	1450	8334	1460

*SI *Sequence identity, *N*m the number of occurrences of the subject motif in the given RK and RLCK classes, *N*c the number of a given class in the dataset according to either the Shiu classification scheme, *P *the total number of the subject motif in the dataset

### Sequence features of NRE segments from LRR-VI-2 class

**Fig. 6:**
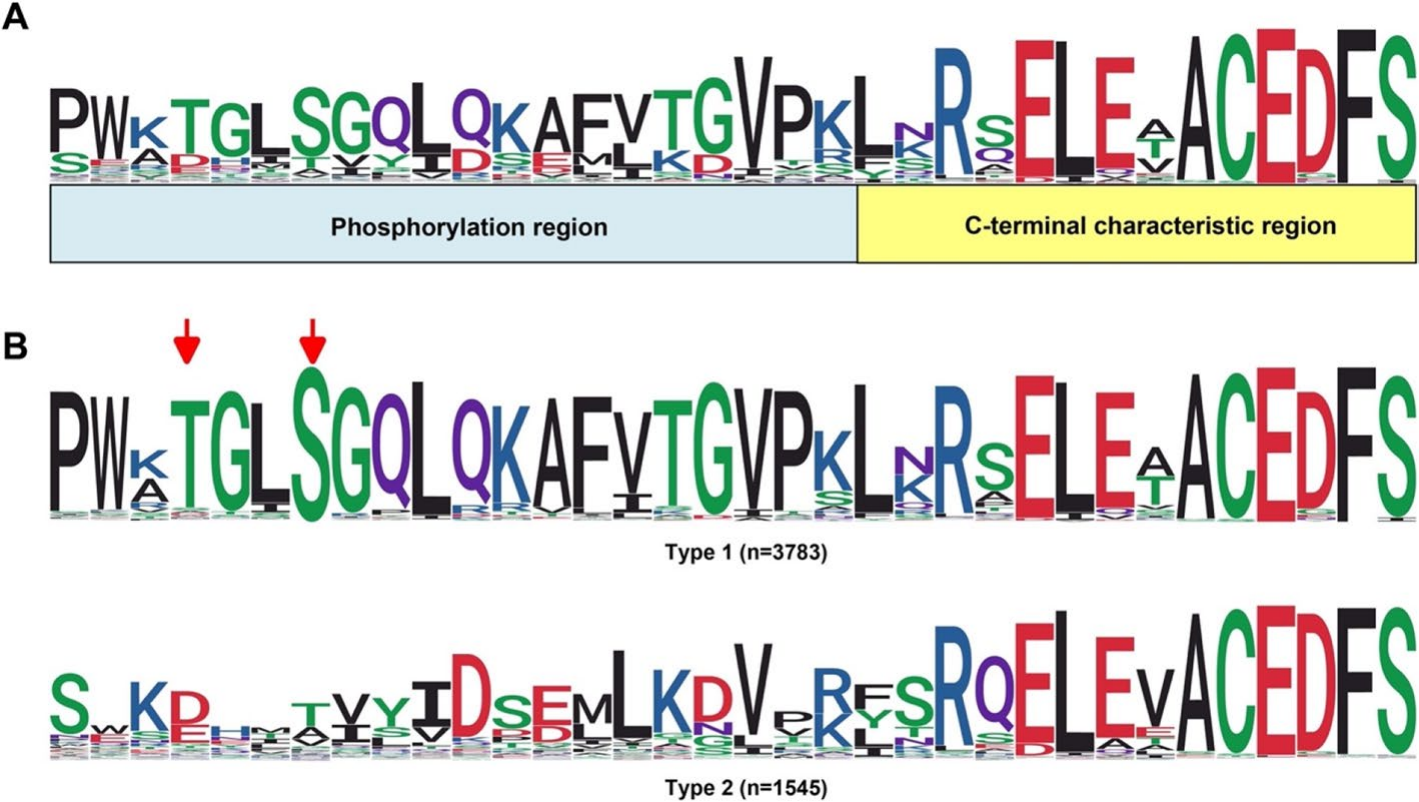
Sequence features of NRE segments in the LRR-VI-2 class. **A **Schematic representation of the NRE sequence characteristic of the LRR-VI-2 class. **B **Two types of NRE segments in the LRR-VI-2 class; (1) Type 1 with two experimentally determined phosphorylation sites at positions − 28 (S) and − 31 (T); (2) Type 2 with no apparent phosphorylation site at these positions. The red arrows indicate the phosphorylation sites

To further investigate the role of the phosphorylation motif in the LRR-VI-2 class, we conducted an analysis of the NRE sequence features, revealing that a subset of LRR-VI-2 displayed a remarkable level of amino acid conservation in the NRE segment. This conserved sequence comprised two distinct regions: a phosphorylation site region and a C-terminal characteristic region (Fig. 6A). The phosphorylation site region contained a serine residue at position − 28 from the C-terminal to the N-terminal, which has been experimentally confirmed to undergo phosphorylation in the EPSD dataset. WebLogo analysis of the LRR-VI-2 NRE sequence revealed two major types of sequences based on the presence or absence of the serine residue at position − 28, which is supported by maximum-likelihood clustering (Figs. 6B, S6). The most common type of LRR-VI-2 NRE sequence (*n* = 3783) featured a conserved motif, ‘VGPWKpTGLpSGQLQKAFVTGVP,’ in phosphorylation region, which contained a single phosphoserine site at -28 S and often had an experimental phosphothreonine site at position − 31T. The other type of NRE segment (*n* = 1545) lacked an apparent serine phosphorylation site at position − 28, and although a putative threonine phosphorylation site was sometimes present, it did not have the phosphorylation motif.

### Phosphorylation of the NRE motif in LRR-VI-2 protein MDIS1 alleviated the auto-phosphorylation of its co-receptor MIK1

**Fig. 7:**
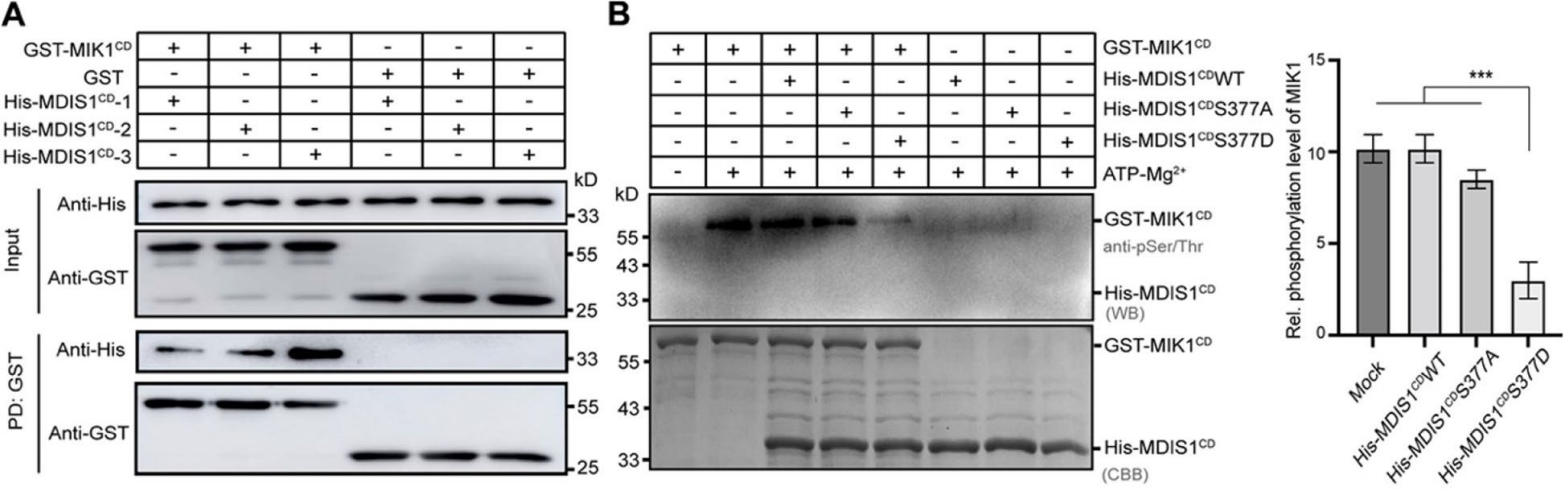
The phosphorylation site of the NRE motif of *Arabidopsis* LRR-VI-2 MDIS1 plays a key role in MIK1 kinase activity. **A **GST pull-down assays. GST-MIK1^CD^ pulled down the tested His-MDIS1. His-MDIS1^CD^-1, -2, and -3 represent three independently repeated GST pull-down experiments. GST protein as the negative control. Proteins in the upper panel were detected using an anti-His antibody, and those in the lower panel were detected using an anti-GST antibody. **B **In vitro kinase assays. GST-MIK^CD^, His-MDIS1^CD^ WT, His-MDIS1^CD^ S377A, and His-MDIS1^CD^ S377D were individually co-expressed with λ-phosphatase in *Escherichia coli* to obtain dephosphorylated proteins. The samples (1 mM) were incubated at 25℃ with 1 mM ATP and 10 mM Mg^2+^ for 1 h. Proteins in the upper panel were detected using an anti-pSer/Thr antibody, and those in the lower panel were stained with Coomassie Brilliant Blue (CBB). The relative phosphorylation level was analyzed using ImageJ. Three replicates were performed, with similar results. Data are presented as the mean ± SD; *** *p*  < 0.001, tested by one-way ANOVA. The term “Mock” refers to the auto-phosphorylation level of MIK1 without incubation with His-MDIS1 proteins

Multiple sequence alignment of the kinase domain revealed that several characteristic kinase residues were not conserved in members of the LRR-VI-2 class (Fig. S7), rendering it a group of pseudokinases with compromised kinase activity. The MALE DISCOVERER1 (MDIS1), an LRR-VI-2 protein, was known to form a heterodimer with MDIS1-INTERACTING RECEPTOR LIKE KINASE1 (MIK1) on the pollen tube cell surface [27]. Therefore, we selected the MDIS1-MIK1 interaction as a model system to verify the functional relevance of the NRE phosphorylation motif in the LRR-VI-2 class. To investigate the functional role of the phosphorylation site in the NRE motif, we recombinantly constructed the cytoplasmic regions of MDIS1 (MDIS1^CD^) and MIK1 (MIK1^CD^) with His and GST tags, respectively (Fig. S8). Interactions between MDIS1^CD^ and MIK1^CD^ were evaluated using GST pull-down assays. The His-MDIS1^CD^ wild-type (WT) interacted with the GST-MIK1^CD^, but not with the GST protein (Fig. 7A). In vitro kinase assays were then performed to assess the phosphorylation activity of His-MDIS1^CD^ and GST-MIK1^CD^. We observed strong auto-phosphorylation of GST-MIK1^CD^ (Lane 1–2), while the self-phosphorylation activity of His-MDIS1^CD^ proteins was weaker or absent (Lane 6–8) (Fig. 7B). Additionally, the internal control showed that myeline basic protein (MBP) could be phosphorylated by GST-MIK1, not by His-MDIS1 (Fig. S9). Furthermore, we investigated whether MDIS1 variants influenced the phosphorylation level of MIK1. Our analysis revealed that incubation of GST-MIK1^CD^ with His-MDIS1^CD^ S377D mutant led to a marked reduction in the level of phosphorylated GST-MIK1^CD^ protein (Lane 5) (Fig. 7B), whereas GST-MIK1^CD^ proteins exhibited a similar phosphorylation pattern when incubated with either His-MDIS1^CD^ WT or His-MDIS1^CD^ S377A mutant (Lane 3–4) (Fig. 7B). These findings suggested that phosphorylation of the NRE motif in MDIS1 was functionally relevant.

## Discussion

### A conserved 14-amino acid sequence forms a characteristic α-helix and ST motif upstream of the core RK and RLCK kinase domain

A consensus sequence, ‘FSYEELEKAT[D/N]NF[S/D]’, located at the C-terminal of NRE segments, appears to be a common feature in the majority of RK and RLCK sequences (Fig. 4D-F), with the exception of nine classes of the RLK family (LRR-III, LRR-VI-2, LRR-VII-1, LRR-XI-2, LRR-XIIIa, LRR-XIIIb, RLCK-II, Singleton, and URK-2) out of the 65 classes defined in the Shiu classification scheme (Fig. S10A). In these nine classes, the C-terminal of NRE segments exhibits highly characteristic features within the same classes, yet appears to be mutually distinctive among different classes, indicating the presence of alternative regulatory mechanisms that deserve further exploration.

Upon surveying the NRE sequences of 510,233 RLKs, it was found that the majority of RK and RLCK possess a similar sequence ‘FSYEELEKAT[D/N]NF[S/D]’, which comprises an α-helix and an ST motif (TxxF) (Fig. 4J, K). This sequence is followed by two other motifs, including an ASX motif and a solvent-accessible Schellman loop [28]. These motifs participate in a complex network of hydrogen bonding interactions along the polypeptide backbone, which forms a long extension that caps the top of the twisted β-sheets in the N-lobe of the core kinase domain in the RKs and RLCKs. The universal presence of the α-helix and ST motif is evident from their occurrence in a series of RK structures, such as BAK1 (3TL8), BIR2 (4L68), BRI1 (4OH4), and FER (7XDX) (Fig. S10B, Fig. 4J). Furthermore, some RLCKs without TM region structures also reveal the same α-helix and ST motif (Fig. S10B, Fig. 4K). For example, the BIK1 (5TOS) with the NKE segment retains the upstream α-helix and ST motif [28]. In human IRAKs, including IRAK1 (6BFN), IRAK3 (6RUU), and IRAK4 (6TI8), all of the N-terminal kinase extension also possess a similar α-helix (αB) and an ST motif characterized by the signature TxxF (Fig. S10B) [29]. While the ASX motif and Schellman loop are absent in IRAK1 and IRAK3, the ST motif can be found in all of them. Moreover, the conservation of α-helix and ST motif in members of the RLK/Pelle superfamily, which includes spanning apicomplexans, insects, and mollusks, suggests that these sequence features could be useful for detecting members of this superfamily.

In contrast, non-RLK/Pelle superfamily kinases, such as MKK1 (MAPK Kinase) ERK activator and Ras of complex protein, which possess a kinase domain but lack the characteristic RLK family sequence ‘FSYEELEKAT[D/N]NF[S/D]’. The structures of Roco kinase (4F0F) or the Raf kinase (6U2G) fail to identify the presence of α-helix and ST motif located upstream of the kinase domain (Fig. S10C). Similarly, other protein kinase families also lack the RLK/Pelle specific α-helix and ST motif, as evidenced by structural representatives from various studies (Fig. S10C).

To explore the functional significance of the characteristic sequence of the RLK family, we focus on the FER protein, which is involved in plant development and stress response, and participates in various physiological processes [30]. FER possesses a typical cytoplasmic kinase domain and the conserved C-terminal NRE sequence, and exhibits both kinase and auto-phosphorylation activities [31]. However, the regulation of its kinase activity by NRE remains inconclusive. Our data suggest that the C-terminal conserved sequence in the NRE segment represses FER kinase activity (Fig. 5). The C‐termini of the NRE segments are also found essential for chitin response in both *At*CERK1 and *Os*CERK1 [22]. In summary, the characteristic α-helix and ST motif forming an N-terminal extension of the core kinase domain is a hallmark of the RLK/Pelle superfamily, while ‘FSYEELEKAT[D/N]NF[S/D]’ and some of its variations with minor classes define the N-terminal boundary of the core kinase domain.

### Comprehensive analysis of phosphorylation motifs in the NRE segment of RKs and RLCKs: Implications for plant signal transduction pathways

Phosphorylation is a reversible post-translational modification, which transfers a phosphoryl group from ATP to specific serine, threonine, or tyrosine residues within their target proteins [32]. For RLKs, most phosphorylation studies mainly focus on their kinase region, and few studies have explored phosphorylation events in the NRE segment. Here, we present the most comprehensive data on phosphorylation motifs in the NRE segment, which are associated with RK and RLCK classes to facilitate targeted phosphorylation analysis of specific sets of proteins. This dataset serves as a valuable reference for inferring the conservation of phosphorylation sites in the NRE segment across plant species.

The NRE segment of RK and RLCK is characterized by a high serine content compared with other parts of their sequence, indicating that it is a region where frequent phosphorylation events occur (Fig. 4D-F). Throughout the phosphorylation motif analysis using experimental data from the EPSD databases, specific motifs were identified that are enriched in particular RK or RLCK classes, while other motifs are distributed widely across different classes (Table S1, Table 1). Phosphorylation mainly occurs on serine and threonine residues in plants, although tyrosine phosphorylation is also observed and accounts for around 5% of all phosphorylation events. This suggests that plant signal transduction may also rely on tyrosine phosphorylation, despite the lack of animal RTK [33]. One example of a protein that undergoes phosphorylation at both serine/threonine and tyrosine residues is the brassinosteroid (BR) receptor kinase BRI1, whose NRE segment is confirmed as an activator kinase domain and determinant of autophosphorylation specificity [34]. Furthermore, some key RK classes are enriched in conserved motifs with experimentally determined phosphorylation sites. For instance, the plant-specific *Cr*RLK1L-1 is an important RLK class in various cellular processes [30]. The NRE segment of the *Cr*RLK1L-1 class is enriched with the motif ‘pTGpSpYApSpSLPpSBLCR’, which is conserved in 479 species and has a similarity above 66.8%. In addition to serine/threonine phosphorylation, this motif also features tyrosine phosphorylation, demonstrating that the *Cr*RLK1L-1 class may function as a dual-specificity kinase. The highly conserved phosphorylation motif in the NRE segment likely plays a significant regulatory role in the transduction of defense signals from cell-surface receptors to downstream signaling components in plants for the *Cr*RLK1L-1 class. Meanwhile, the motifs from algae referred mainly to cellular energy signaling, plant defense signal transduction, and environmental stimuli response.

The motif ‘VGPWKpTGLpSGQLQKAFVTGVP’ found in the LRR-VI-2 class is highly conserved and widely distributed across plant species, and coincides with a known phosphorylation site, suggesting its importance in regulating the plant signal transduction pathway. Consensus sequence analysis further reveals that two phosphorylation sites (-28 S and − 31T) in the LRR-VI-2 class are highly conserved, suggesting a potential synergistic mechanism (Fig. 6). Additionally, our motif analysis has identified novel and conserved motifs containing S/T/Y residues that are specific to the RK and RLCK classes but have not been reported in NRE phosphorylation events. By characterizing the sequence motifs of NRE segments with phosphorylation sites, it may be possible to develop a predictive algorithm that can differentiate between S/T/Y residues that are more likely to be phosphorylated from those that are less likely to be phosphorylated in the NRE segments of RKs and RLCKs. In conclusion, the study of conserved features of phosphorylation sites in NRE segments can provide valuable insights into the molecular mechanisms underlying plant signal transduction.

### Phosphorylation sites in NRE segments of RLKs function as a ‘kinase switch’ to regulate downstream kinase activity: evidence from LRR-VI-2 class

Recent research suggests that the phosphorylation sites located upstream of the C-terminal NRE sequence, which is a characteristic feature of RKs and RLCKs, function as an on/off switch to alternate the conformation of the downstream core (pseudo) kinase domain [35]. For example, an unphosphorylated NRE segment of RK ACR4 in *Arabidopsis* preferentially interacts with a kinase domain near the activation loop, which presumably holds the kinase domain in an inactive state [36]. In contrast, phosphorylation of this NRE segment can cause it to interact preferentially with the N-terminal lobe of the kinase domain, resulting in an ACR4 variant with a defect in substrate phosphorylation [36]. Another example is RK BAK1, which transforms CERK1 from an inactive to a primed state by inducing an allosteric change through mediating NRE phosphorylation [23]. Thus, it can be speculated that the phosphorylation region of the NRE segment is essential for kinase activity in RLKs.

We propose a schematic functional division of the NRE segment in LRR-VI-2 that can be applied to most NRE segments (Figs. 3A and 6A). Although NRE segments are generally considered intrinsically disordered and prone to significant sequence variability, the NRE segment of one group of LRR-VI-2 is remarkably conserved in composition. Furthermore, the length of this conserved sequence falls within the typical length range for an NRE sequence (Figs. 3A, S1A). Notably, the motif ‘VGPWKTGLSGQLQKAFVTGVP’, which was characterized from LRR-VI-2 based on a total of 3532 NRE segments from 490 plant species, is the most conserved and contains an experimentally-determined phosphorylation site ‘TGL(pS)GQLQK’ located at position − 28 (Table 1; Fig. 6B). Additionally, members of LRR-VI-2 are atypical kinases with variations in the glycine-rich loop and the VAIK/HRD/DFG motifs in the catalytic domain that are essential for catalysis [37] (Fig. S7).

MDIS1 is a well-studied member of the LRR-VI-2 class, which forms a receptor heteromer with MIK1 on the surface of pollen tube cell [27]. In a phosphorylation assay, an aspartic acid mutant S377D in MDIS1, which mimics the effect of phosphorylation, was found to compromise the kinase activity of MIK1, suggesting the presence of an exact phosphorylation regulation in NRE segment (Fig. 7). This could be due to either allosteric regulation or scaffolding molecules in cell signaling [38, 39]. Allosteric regulation may require dynamic transitions between different conformational states, as seen for canonical kinases [40, 41]. Alternatively, pseudokinase domains might be more static than other kinase domains, allowing them to serve as rigid scaffolds. The common presence of serine in the LRR-VI-2 pseudokinases subtype suggests its crucial role in the activation process, despite the variations of essential residues in the kinase domain. These results indicate that the phosphorylation site (position − 28) of the NRE segment of *Arabidopsis* LRR-VI-2 MDIS1 is necessary to regulate MIK1’s kinase activity.

The relatively short NRE segments provide a minimal set of functional elements that coordinate with each other to complete its task as a ‘kinase switch’. While the characteristic C-terminal α-helix and ST motif are universal to all RKs and RLCKs, the LRR-VI-2 model suggests a plausible mechanism through which phosphorylation in the NRE motif regulates downstream kinase activities in the RLK family. The ‘kinase switch’ may control the kinase activity either directly or allosterically through its binding partners upon phosphorylation of the NRE segment.

## Materials and methods

### Plant genomes used for RK and RLCK sequences identification

Plant genomes containing RK and RLCK for NRE analysis were obtained from several sources, including genomes of 84 plant species from the Joint Genome Portal (JGI, https://genome.jgi.doe.gov/portal/); 55 species from The Genome Warehouse (GWH) [42]; 45 species from Ensembl Plants [43]; 53 species from the NCBI database (https://www.ncbi.nlm.nih.gov/); 29 species from the Amazon Web Services (AWS) (https://uc3-s3mrt1001-prd.s3.us-west-2.amazonaws.com/); 32 species from the GigaDB database (http://gigadb.org/site/index); 30 species from the ePlant database (http://eplantftp.njau.edu.cn/); 14 species from the Medicinal Plant Genomics Resource (MPGR, http://mpgr.uga.edu/); 14 species from the Hardwood Genomics Project (HGP, https://www.hardwoodgenomics.org), and an additional 172 species from other databases. References and relevant information regarding this non-redundant set of 528 species and their source are available in Supplemental Data (Table S2). The gene domain organization and gene structure for plant genomes from GenBank and RefSeq in NCBI were predicted using the EST-based method or transcript-to-genome sequences. In addition, some recently published genomes also used de novo prediction and homology-based methods for prediction. The 528 species were divided into seven types, including Algae, Charophytes, Bryophytes, Lycophytes, Ferns, Gymnosperms, and Angiosperms. Each type includes one or more clades, with Algae containing red algae, brown algae, and green algae; Bryophytes containing liverworts and mosses; Gymnosperms containing ginkgo and conifers; and Angiosperms containing basal angiosperms, monocots, and eudicots.

### Identification of NRE sequences in the RLK family

The genome sequences of the 528 plant species were used to identify and classify RKs and RLCKs sequences using a set of pre-compiled HMMs [8], implemented in the iTAK program [44]. RKs and RLCKs designation and classification are obtained using the latest Shiu classification scheme according to the plant protein kinase phylogeny and extracellular domain identities [8]. In addition, the transmembrane region of each RK and RLCK sequence was identified using a consensus of the outputs of the TMHMM [45] and Phobius [46] analysis. The N-terminal boundary of each NRE segment was defined as the residue immediately following the C-terminal residue of the transmembrane sequence. The boundary of the cytoplasmic kinase domain was identified using Pfam [47] with two Pfam families: PF00069 referring to “PKinase” and PF07714 referring to “PK_Tyr_Ser-Thr.” Inspection of the HMM profiles for both Pfam families revealed a characteristic (L/I/V)GXG motif between positions 5–8 at the N-terminal of the kinase domain. The NRE C-terminal boundary was defined immediately before the N-terminal residue of the kinase domain. All NRE sequences and associated information were imported into a custom PostgreSQL database for further analysis.

### Analysis of NRE sequences

The length and composition of the NRE were calculated using a custom Python script, which allowed plotting either the whole dataset or its subset according to the RK and RLCK classes or the types of plant species. WebLogo [48] analyzes the specific distribution of amino acid profiles at each position. Amino acid profiles are constructed using the first 20 amino acids of the NRE aligned to the N-terminal and the last 20 amino acids of the NRE aligned to the C-terminal. Hydrophobic, polar, acidic, basic, and neutral residues are shown in black, green, red, blue, and purple in the WebLogo profile, respectively.

### Measurement of kinase activity

We assessed kinase activity using the Kinase-LumiTM Luminescent Kinase Assay Kit (Beyotime, China, S0150S) [49]. The assay kit provides a method for quantifying kinase activity by measuring the luminescence generated from the remaining ATP in solution after a kinase reaction. The luminescent signal is directly proportional to the residual ATP concentration and inversely proportional to the kinase activity. The wild-type cytoplasmic domain of FERONIA (FER) (residues 518–816) and its corresponding mutant (residues 518–816, K565R) were cloned into modified pRSFDuet-1. The kinase domain of FER (residues 536–816), which did not contain the 14-amino acids in the C-terminal of the JM segment, is cloned into pET-28a. The recombinant proteins were expressed and purified following a protocol described previously [50]. Specifically, 1 µM proteins were incubated in 50 µL kinase reaction mixture containing 50 mM HEPES buffer, pH 7.5, 10 mM MgCl_2_, 10 mM MnCl_2_, 1 mM EGTA, 10 µM ATP, followed by the addition of 50 µL Kinase-Lumi Chemiluminescence Kinase Detection Reagent. Following reaction at room temperature for 10 min, ATP consumption (µM), representing the kinase activity per 1.0 µM protein, was measured by chemiluminescence detection using a multiscan spectrum (Thermo scientific, Fluoroskan Ascent™ FL). The assay was standardized against an ATP standard curve.

### Motif discovery and analysis

Based on the WebLogo analysis, the 14 C-terminal residues were removed before motif analysis. Motif analysis was conducted with the middle part of the NRE segments using the MEME Suite, including MEME for motif discovery and FIMO for motif identification. MEME was used to predict the potential motifs with a *p*-value threshold of 0.005 using an iterative statistical algorithm [51], while FIMO was used to identify the NRE sequences containing these motifs with a *p*-value threshold of 0.001 [52].

The normalized frequency of each motif was calculated using the formula F = $\frac{{{N}_{m}}^{2}*{N}_{total}}{P*{Nc}^{2}}$ ($\frac{{N}_{m}}{P\times {A}_{c}}*\frac{{N}_{m}}{Nc}$), in which *N*_*m*_ was the number of occurrences of the subject motif in the given class according to the Shiu classification scheme, *P* was the total number of the subject motif in the dataset, and *A*_*c*_ was the abundance score of a given class in the dataset according to the Shiu classification scheme. The abundance score was calculated as *A*_*c*_ = $\frac{{N}_{c}}{{N}_{total}}$, in which *N*_*c*_ was the number of a given class in the dataset according to either the Shiu classification scheme or the types of plant species defined above, *N*_*total*_ is the total number of NRE sequences in the dataset. Motifs with a normalized frequency higher than 1 were selected for further analysis. A normalized frequency higher than 1 indicated an overrepresented class for the given motif, while a normalized frequency lower than 1 indicated an underrepresented class for the given motif.

### Identification of phosphorylation sites in motifs of the NRE segments

Our study utilized experimentally determined phosphorylation data from 23 plant species to investigate the relationship between the NRE segment and phosphorylation motifs (Table S3). The genomes of 23 plant species collectively contained 35,913 NRE segments from 36,912 RKs and RLCKs. We mapped the motifs identified in the previous step onto the NRE segments in the RLK and RLCK sequences using FIMO. From the EPSD database [53], a list of experimentally determined phosphorylation sites was obtained. The starting and ending positions of the phosphorylation sites were compared within the boundary of FIMO motifs in the NRE segments to obtain a list of phosphorylation sites entirely contained in an identified motif. Motifs without serine/threonine/tyrosine residues at the consensus sequence at the expected phosphorylation site were excluded from further analysis. To investigate the consensus analysis of the identified motif sequence and the matching NRE sequence, we used the formula I =$\frac{{N}_{Im}}{{N}_{m}}$, in which *N*_*Im*_ is the number of consensus amino acids in the motif sequence and the NRE sequence, and *N*_*m*_ is the number of amino acids in the matching NRE sequence. The motif having more than 80% identity with the matching NRE sequences was considered highly conserved and similar. Motifs with sequence identity between 50% and 80% were considered moderately conserved and similar. The distributions of these motifs in various species were analyzed using R. In this analysis, 457,685 NRE segments without domain were analyzed except 67,263 NRE segments with domain.

### Sequence alignment

The representative sequences of LRR-VI-2 class cytoplasmic domains were retrieved from the abovementioned dataset. Multiple sequence alignment (MSA) was performed using MAFFT v7 [54] with the L-INS-I settings, with figures prepared using Jalview 2.11.1.4.

### Expression and purification of MDIS1 and MIK1 protein

The cytoplasmic kinase domains of MALE DISCOVERER1 (MDIS1, residues 362–668) and MDIS1-INTERACTING RECEPTOR LIKE KINASE1 (MIK1, residues 698–983) were cloned into modified pRSFDuet-1 and pGEX-6P-1 vectors containing an N-terminal 6×His tag and N-terminal glutathione S-transferase (GST) tag, respectively. We constructed two MDIS1 mutants (S377A and S377D) to investigate the functional role of the phosphorylation site in the positions of serine − 28 of the NRE segment. Mutants with aspartic acid (D) and alanine (A) at the − 28 position were designed to mimic serine’s phosphorylation and dephosphorylation states, respectively. The fused proteins were expressed in the chemically competent *E. coli* BL21 (DE3) from TransGen Biotech. Cells were grown to an OD_600_ value of 0.6–0.8 at 37 °C and then induced with 0.5 mM Isopropyl β-D-thiogalactopyranoside (IPTG) followed by 18–20 h incubation at 16 °C. MIK1 and MDIS1 were co-expressed with the λ-phosphatase for dephosphorylation. The cells were harvested by centrifugation at 4,200 rpm for 15 min, resuspended with 30 mL lysis buffer containing 20 mM Tris-HCl (pH 7.5), 150 mM NaCl, and 10 mM imidazole, and homogenized using a low-temperature ultra-high pressure cell disrupter (JNBIO, Guangzhou, China). The supernatants were collected after centrifugation at 12,000 rpm for 1 h at 4 °C. The His-MDIS1^CD^ protein was loaded onto a Ni-NTA agarose column (GE Healthcare) and eluted using elution buffer containing 20 mM Tris-HCl, 150 mM NaCl, pH 7.5, and 300 mM imidazole, and then exchanged into Tris buffer without imidazole. The GST-MIK1^CD^ was loaded onto GST agarose (Glutathione Sepharose 4B, GE Healthcare) and eluted using GST elution buffer containing 50 mM Tris-HCl, 20 mM glutathione, 150 mM NaCl, pH7.5.

### GST pull-down assay

The purified GST-MIK1 was initially combined with GST agarose for 4–6 h, followed by three washes with GST binding buffer. Then, the purified His-MDIS1^CD^ wild-type (WT) was mixed with the MIK1-GST proteins and incubated for 6 h at 4 °C in GST binding buffer (20 mM Tris-HCl, pH 7.5, 150 mM NaCl, 5% (vol/vol) glycerol). The beads were collected by centrifugation and washed five times with buffer containing 20 mM Tris-HCl, pH 7.5, 150 mM NaCl, 0.3% Triton X-100, 0.1% SDS. Finally, the proteins bounded on the beads are boiled with 1× SDS loading buffer in 95 °C water bath and analyzed by both SDS-PAGE and immunoblotting with anti-GST (ABclonal, AE001) and anti-His (ABclonal, AE003) antibody.

### In vitro MDIS1 and MIK1 phosphorylation assays

Protein kinases play a crucial role in catalyzing the transfer of a γ-phosphate group from ATP to Serine or Threonine side chains on protein substrates. The in vitro phosphorylation assay in this study utilizes specific anti-phosphoserine/phosphothreonine antibody-based detection on membrane in Western blotting for the analysis of phosphorylated proteins. The experiment followed methods described previously [31]. MIK1 and MDIS1 proteins were co-expressed with the λ-phosphatase for dephosphorylation. For phosphorylated assays, each of the His-MDIS^CD^ WT, His-MDIS^CD^ S377D, and His-MDIS^CD^ S377A was co-incubated with GST-MIK1^CD^ at 25℃ for 1 h in assay buffer containing 25 mM Tris-HCl at pH 7.5, 10 mM MgCl_2_, 1 mM ATP. The reaction was stopped by adding 1× SDS loading buffer. The proteins were separated by 12% SDS-PAGE and immunoblotting. The phosphorylated GST-MIK1^CD^ and His-MDIS^CD^ proteins were detected using an antibody against pSer/pThr (ABclonal, AP0893). In addition, the presence of the two recombinant proteins was verified by Coomassie blue G250. The relative phosphorylation level was analyzed using ImageJ. Three replicates were conducted. The data are presented as the mean ± SD; ***p < 0.001, determined through one-way ANOVA.

## Conclusion

By application information theory and data mining techniques, we conducted a comprehensive analysis of 510,233 NRE sequences in RLKs from 528 plants. Additionally, we performed experimental investigations utilizing recombinant RKs to explore the function of the NRE in vitro. This study provides a rich dataset that can be used to investigate NRE segments within individual RLKs, thereby enhancing our understanding of the underlying mechanisms involved in RLK signal transduction. Our findings reveal that the majority of NRE segments exhibit a serine-rich region and a 14-amino-acid consensus sequence. Notably, this consensus sequence encompasses a characteristic α-helix and ST motif, which form an N-terminal extension of the core kinase domain, a distinctive feature of the RLK/Pelle superfamily. Furthermore, we identified 29 novel motifs with highly conserved phosphorylation sites. In particular, phosphorylation of an NRE motif in MDIS1, an LRR-VI-2 member, was found to modulate the auto-phosphorylation of its co-receptor, MIK1, suggesting the potential role of NRE as a ‘kinase switch’ in RLK activation.

### Supplementary Information

**Supplementary Material 1.**

## Data Availability

The 510,233 NRE sequences of RKs and RLCKs from 528 plant species, all NRE sequences that match these phosphorylation motifs, and the program codes supporting the findings of this study are available from http://rlktm.biocloud.top/.

## Abbreviations

RLKs: Receptor-like kinases
RTK: Receptor tyrosine kinase
RKs: Receptor kinases
RLCKs: Receptor-like cytoplasmic kinases
ER: Extracellular region
ECD: Extracellular domain
SP: Signal peptide
TM: Transmembrane
CR: Intracellular region
JM: Juxtamembrane
KD: Kinase domain
NKE: the N-terminus of the kinase domain
NRE: the N-terminal regulatory elements of the kinase domain
EST: Expression Sequence Tag
PTMs: Post-translational modifications
IRAKs: Interleukin Receptor-associated Kinases
LRR: Leucine-rich repeat
SLGs: Self-incompatibility-locus glycoproteins
ErbB-1: Epidermal growth factor
EphB2: Eph receptor kinase
TGF-β: Type I transforming growth factor
FER: FERONIA
SI: Sequence identity
MDIS1: Male Discoverer1
MIK1: MDIS1-Interacting Receptor Like Kinase1
PRK4: Pollen Receptor Like Kinase 4
BIK1: Botrytis-Induced Kinase1
WT: Wild-type
BR: Brassinosteroid
